# Construction of the SHP-GLOX lignin regulation system and its application in rice straw

**DOI:** 10.1186/s13007-022-00917-8

**Published:** 2022-06-18

**Authors:** Qingdong Wang, Jiayuan Zhang, Yan Li, Ran Wang

**Affiliations:** 1 Henan Key Laboratory of Bioactive Macromolecules, Laboratory of Straw Enzymatic Technology Research, College of Life Science, Zhengzhou University, Zhengzhou, 450001 Henan China; 2 College of Life Science, Henan Agricultural University, Zhengzhou, 450002 Henan China; 3 Department of Clinical Medicine, Nan Yang Medical College, Nanyang, 473000 Henan China

**Keywords:** *Agrobacterium*-mediated transformation, Lignin, Soybean hull peroxidase, glyoxal oxidase, Transgenic rice, Fermentation

## Abstract

**Background:**

There is great productivity of rice(*Oryza sativa* L. spp. *japonica*) straw in China, which is a potential source of biomass for biofuel and forage. However, the high levels of lignins in rice straw limited its usage and induced the formation of agricultural waste. In order to modify the lignins contents to improve biofuel production and forage digestibility, we selected Soybean hull peroxidase (SHP) and Glyoxal oxidase (GLOX) as candidate genes to improve quality of rice straw. SHP, a class III plant peroxidase, is derived from multiple sources. It has several advantages, such as high resistance to heat, high stability under acidic and alkaline conditions, and a broad substrate range. SHP is speculated to be useful for lignin degradation. Glyoxal oxidase (GLOX) is an extracellular oxidase that can oxidize glyoxal and methylglyoxal in the extracellular medium to generate H_2_O_2_.

**Results:**

In the present study, the *SHP* and *GLOX* genes in pCAMBIA3301-glycine-rich protein (GRP)-SHP-GLOX, designated the K167 vector, were optimized and introduced into rice embryos using *Agrobacterium*-mediated transformation. Positive transgenic rice embryos were examined using molecular, physiological, biochemical and fermentation tests. The outcomes suggested that SHP degraded lignin effectively.

**Conclusions:**

This research has created a rice breeding material with normal growth and yield but stalks that are more amenable to degradation in the later stage for use in breeding rice varieties whose stalks are easily used for energy. Our results will improve the industrial and commercial applications of rice straw.

## Background

China produces rice (*Oryza sativa* L. spp. *japonica*) straw in large quantities, providing a crucial source of biomass for raw materials. The straw primarily contains cellulose, hemicellulose, and lignin. However, lignin has a complex structure that makes its degradation difficult and, in turn, limits the utility of rice straw. Lignin is a highly abundant and renewable resource across the globe that holds great potential for the production of value-added chemicals [1–3]. China produces lignocellulosic resources in large quantities in the form of agricultural waste, industrial residues, and residual wood. Crop straw accounts for a large proportion of lignocellulosic agricultural resources in agriculture-based countries; in addition, it is widely used as a raw material for animal feed, fertilizer, alkali materials and energy production [4].

The precise underlying mechanism of lignin biosynthesis remains unknown. However, according to previous studies, broadly, it involves three steps: shikimate metabolism, phenylpropane metabolism, and lignin synthesis pathways. Lignin production or contents can be altered by regulating the expression of related enzymes, which can be achieved via genetic engineering [5–7]_._

Lignin is formed from peroxidase-catalysed polymerization of lignin monomers. Peroxidase is an oxidoreductase enzyme that utilizes hydrogen peroxide (H_2_O_2_) to oxidize organic compounds. It plays a crucial role in a myriad of physiological and biochemical processes in plants, such as photosynthesis, respiration, disease resistance, and biotic-abiotic stress responses [8]. Peroxidases are ubiquitous in plants, animals, and microorganisms. In addition, peroxidases have found wide applications in multiple areas, such as biodegradation, sewage treatment, biocatalysis and biosensors. Horseradish peroxidase is a well-known commercial enzyme. Soybean hull peroxidase (SHP), a class III peroxidase, is derived from the soybean seed coat, a byproduct generated during the industrial oil production process. SHP has multiple substrates, is resistant to high temperature [9, 10], and functions over a broad pH range, which makes it a valuable research tool [11]. Glyoxal oxidase (GLOX) is an extracellular oxidase that can oxidize glyoxal and methylglyoxal in the extracellular medium to generate H_2_O_2_. As a source of physiological H_2_O_2_, GLOX is also an important class of enzymes involved in the degradation of lignin.

Transgenic technology has been employed to enhance the degradation of straw, which has reduced the cost of feed production and environmental pollution [12]. SHP is used to treat phenol-containing wastewater released by industrial pulping processes, as SHP effectively degrades the lignin present in this wastewater [10]. Thus, lignin degradation has crucial implications in bioremediation and other research investigations [13]. In the current study, we constructed a K167 vector containing the *SHP* and *GLOX* genes and introduced it into the rice genome through *Agrobacterium*-mediated transformation to generate transgenic rice plants with enhanced lignin degradation.

## Results

### Vector construction

**Fig. 1:**
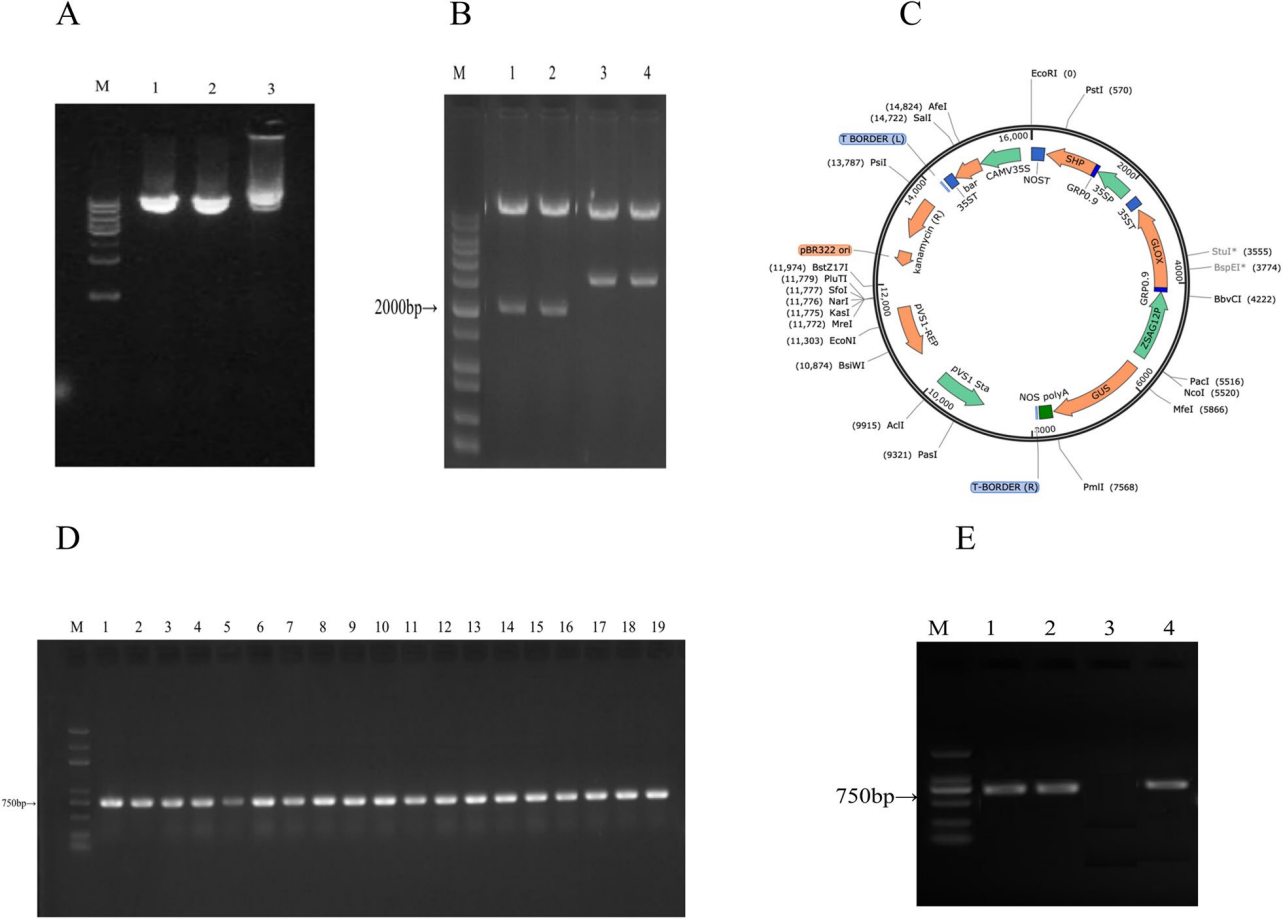
Vector construction and PCR-based identification. **A** Electrophoretic identification of the K167 plasmid. M: Star Marker (DL 2000 Plus); 1–33: Recombinant plasmid to be tested. **B** Double-enzyme digestion verification of the recombinant plasmid. M: 1 kb *Plus; 2–3: NcoI/PmlI enzyme digestion; 4–5: PstI/StuI* enzyme digestion. **C** Schematic diagram of the recombinant plasmid GRP-SHP-GLOX (K167). **D** PCR identification of transgenic rice. M: Star Marker Plus (D2000 Plus); 1–19: K1—K19. (E). PCR identification of transgenic rice. M: Star Marker (D 2000); 1–2: K20—K21; 3: WT; 4: Plasmid K167

To verify the successful construction of the K167 vector, first, the release of the insert was verified by electrophoresis (Fig. 1A) and double enzyme digestion (Fig. 1B). Subsequently, K167 was sent to Shanghai Bioengineering Co., Ltd. for sequencing. The sequencing results combined with the results of enzyme digestion proved that K167 vectors had been successfully constructed, and these vectors were used for subsequent Agrobacterium transformation.

### PCR-based screening of transgenic rice plants

PCR was employed to validate the transgene insertion using SHP-F and SHP-R primers. The amplified PCR product of the 750 bp band size validated the *SHP*-positive status of the transgenic rice plants. The products of the appropriate size were recovered and sent to Shanghai Bioengineering Co., Ltd. for sequencing. The sequencing results correctly verified the identity of the gene fragments, indicating that the genomes of these strains all carried foreign genes and confirming that these 21 strains were positive transgenic plants (Fig. 1D, E).

### Southern blot analysis of transgenic rice plants

**Fig. 2:**
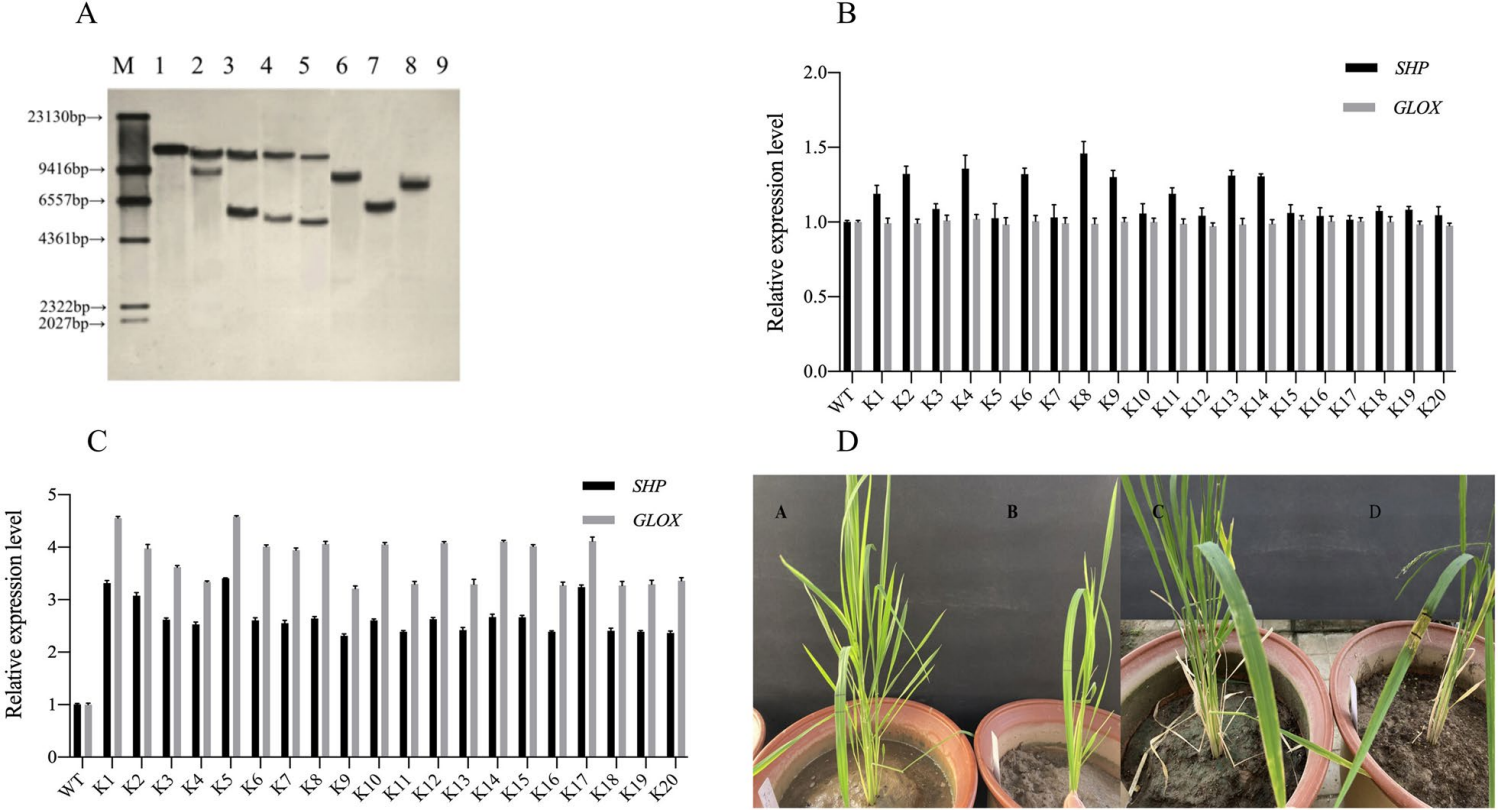
Southern blot, RT–qPCR and Basta smearing identification of rice. **A** Southern blot detection of rice. M: marker; 1: positive control; 2–8: transgenic rice K1—K7; 9: negative control. **B** The expression levels of SHP and GLOX genes in rice at the flowering stage. **C** The expression levels of SHP and GLOX genes in rice at the maturity stage. **D** Basta smear experiment on rice leaves. A, C: Transgenic rice before and after smear; B, D: Wide-type rice before and after smear

Southern blotting confirmed the site of transgene integration. To extract pure rice leaf DNA, repeated extractions using RNase A were performed to eliminate RNA contamination. Lane 1 was the positive control, and the target gene fragment was melted and combined with the probe, so this lane had only one band and its colour was darker. Lane 9 was the negative control and showed no band. K1, K2, K3, and K4 all showed two bands in the lanes, so it can be determined that the number of copies of the target gene contained in the genomes of these four lines was double. K5, K6, and K7 all show a single band in the lanes, so single copies of the target gene were contained in the genomes of these three lanes, indicating a single copy line. The Southern blot results showed that exogenous genes were integrated into the rice genome (Fig. 2A).

### Real-time fluorescent quantitative PCR (RT–qPCR) of transgenic rice plants

RT–qPCR validated the presence of the *SHP* gene in transgenic rice plants. *GAPDH* was used as an internal reference gene, and cDNA from the wild-type rice line was used as a negative control. RT–qPCR detection was performed on the positive transgenic rice plants, and the results proved that the exogenous gene had been transcribed and expressed normally in rice, and the expression level showed certain differences in different periods. The *SHP* gene of transgenic rice was upregulated during the flowering and maturity stages. *GLOX* gene expression was normal during the flowering period and was significantly upregulated during the maturity period (Fig. 2B, C).

### Basta smear experiment

The *Bar* gene in the K167 vector serves as a screening marker, and it imparts resistance against the herbicide Basta, also known as glufosinate. *Bar* gene expression in transgenic rice was analysed using Basta. The resistance of transgenic rice plants to Basta demonstrated *Bar* gene expression (Fig. 2D). Conversely, negative control plants were found to be sensitive to Basta, as they did not express the *Bar* gene.

### Measurement of the agronomic traits

**Fig. 3:**
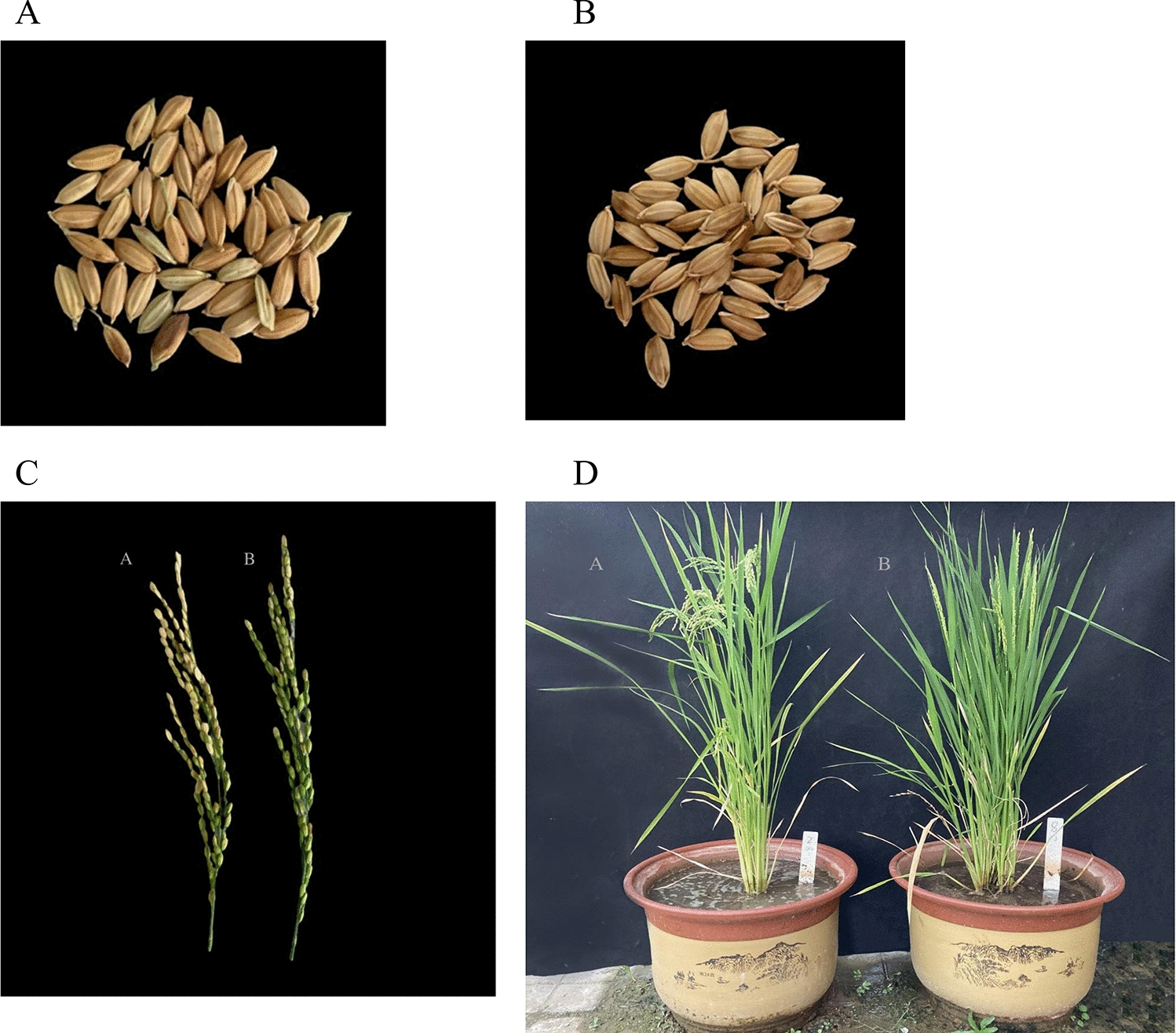
Phenotypes of transgenic rice and wild-type rice. **A** Phenotypes of grains of transgenic rice. **B** Phenotypes of grains of wild-type rice. **C** Comparison of the panicle types between wild-type rice and transgenic rice. A: Wide-type rice; B: transgenic rice. **D** Comparison of the shapes of wild-type rice and transgenic rice. A: Wild-type rice of the control group; B: transgenic rice

As shown in Fig. 3A–D, the wild-type and transgenic rice both grew well and developed normally. There were no significant differences in grain morphology, panicle shape or whole plant morphology between the wild-type and K-series transgenic rice lines.

The agronomic data of the transgenic lines are shown in Table 1, and the agronomic data of the wild-type are shown in Table 2. In terms of plant height, the wild type was not significantly different from K8, K18, and K19 (*P* > *0.05*) but was significantly different from the other strains (*P* < *0.05*). In terms of ear length, the wild type and K1, K4, K7, K8, K9, K10, K12, K14, K15, K16, and K18 were not significantly different (*P* > *0.05*) and were significantly different from other strains (*P* < *0.05*). In terms of the number of tillers, the wild type was not significantly different from K2, K3, K5, and K13 (*P* > *0.05*) but was significantly different from the other strains (*P* < *0.05*). In terms of total grains per ear, the wild type showed no significant difference from K1, K4, K5, K7, K8, K16, K18, and K20 (*P* > *0.05*) and a significant difference from the other strains *(P* < *0.05*). In terms of the number of grains per panicle, the wild type was not significantly different from K1, K4, K5, K7, K8, K11, K16, and K20 (*P* > *0.05*) or significantly different from the other strains (*P* < *0.05*). In terms of the seed setting rate, there was no significant difference between wild-type and all transgenic rice (*P* > *0.05*). In terms of 1000-seed weight, the wild type was significantly different from K2, K3, K6, K9, and K12 (*P* < *0.05*) but not significantly different from the other strains (*P* > *0.05*).

**Table 1:** Agronomic traits of transgenic rice and wild-type rice

Strain	Plant height(cm)	Panicle length(cm)	Tiller number	Total panicle grains	Panicle grains	Seed setting rate(%)	Thousand—grain weight(g)
K1	73.4 ± 1.0^e^	12.9 ± 0.5^a^	12.6 ± 0.7^def^	163.67 ± 5.03^ fg^	145.33 ± 4.04^ghi^	88.81 ± 1.10^c^	29.37 ± 0.74^cde^
K2	66.4 ± 1.5^j^	10.9 ± 0.8^abc^	15.9 ± 0.8^a^	182.33 ± 5.13^ab^	163.33 ± 3.51^bcd^	89.60 ± 1.72^c^	30.72 ± 0.43^ab^
K3	65.6 ± 0.5^j^	10.7 ± 0.7^de^	15.6 ± 0.7^a^	187.67 ± 2.52^a^	174.00 ± 2.65^a^	92.74 ± 2.42^a^	31.30 ± 0.56^a^
K4	77.4 ± 1.2^d^	12.6 ± 0.7^e^	12.2 ± 0.4^defg^	169.67 ± 4.04^cdef^	155.33 ± 3.21^def^	91.56 ± 0.54^abc^	29.50 ± 0.72^cde^
K5	68.6 ± 1.3^i^	11.9 ± 1.3^abcd^	14.1 ± 0.7^bc^	168.33 ± 6.11^def^	151.00 ± 3.46^efg^	89.73 ± 1.49^bc^	29.53 ± 0.74^cde^
K6	77.0 ± 1.2^d^	11.3 ± 0.5^bcde^	12.1 ± 1.5^defg^	182.33 ± 3.51^ab^	167.00 ± 5.29^ab^	91.58 ± 1.49^abc^	30.87 ± 0.35^ab^
K7	78.4 ± 1.4^ cd^	12.6 ± 0.7^cde^	8.1 ± 0.9^i^	164.00 ± 4.36^ fg^	148.67 ± 5.13f^gh^	90.64 ± 0.87^abc^	29.03 ± 0.35^def^
K8	68.8 ± 0.5^hi^	12.4 ± 1.3^abcd^	13.5 ± 0.3^ cd^	161.00 ± 7.21^ fg^	146.67 ± 6.66^fghi^	91.10 ± 0.83^abc^	28.67 ± 0.78^def^
K9	85.6 ± 0.6^a^	13.5 ± 0.8^ab^	8.1 ± 0.9^i^	180.33 ± 3.06^ab^	165.33 ± 4.73^bc^	91.67 ± 1.53^abc^	30.80 ± 0.70^ab^
K10	78.2 ± 1.1^d^	12.5 ± 0.9^abcde^	11.4 ± 0.9^efg^	178.33 ± 2.08^bc^	162.67 ± 5.51^bcd^	91.20 ± 2.07^abc^	30.30 ± 0.70^abc^
K11	70.7 ± 1.5^f^	11.4 ± 1.0^cde^	12.5 ± 0.3^def^	177.00 ± 4.58^bcd^	157.33 ± 3.79^cde^	88.89 ± 0.47^c^	29.23 ± 0.31^cde^
K12	72.7 ± 0.6^ef^	13.7 ± 0.8^a^	11.2 ± 0.9^ fg^	183.00 ± 4.00^ab^	163.33 ± 1.53^bcd^	89.27 ± 1.55^c^	30.80 ± 0.82^ab^
K13	68.2 ± 1.3^i^	11.2 ± 0.8^cde^	14.2 ± 0.4^bc^	155.67 ± 6.03^ g^	140.00 ± 4.58^i^	89.95 ± 0.63^abc^	28.73 ± 0.78^def^
K14	77.6 ± 0.7^d^	13.0 ± 0.5^abc^	11.6 ± 0.8^efg^	156.33 ± 4.73^ g^	140.67 ± 6.03^hi^	89.96 ± 1.79^abc^	28.83 ± 0.23^def^
K15	80.2 ± 1.0^c^	12.7 ± 0.8^abc^	10.8 ± 0.4^ g^	176.67 ± 2.52^bcd^	161.67 ± 1.53^bcd^	91.53 ± 1.92^abc^	29.30 ± 0.60^cde^
K16	77.2 ± 1.5^d^	12.5 ± 0.9^abcde^	12.6 ± 1.4^de^	167.33 ± 6.03^ef^	150.67 ± 3.06^efg^	90.08 ± 1.61^abc^	28.80 ± 0.61^def^
K17	72.3 ± 1.3^efg^	11.6 ± 1.2^cde^	12.2 ± 0.4^defg^	155.67 ± 6.11^ g^	142.00 ± 6.56^hi^	91.20 ± 0.67^abc^	28.00 ± 0.46^e^
K18	82.5 ± 1.0^b^	12.5 ± 0.9^abcd^	8.4 ± 0.7^hi^	175.67 ± 6.81^bcde^	162.33 ± 3.06^bcd^	92.46 ± 1.90^ab^	29.80 ± 0.66^bcd^
K19	71.5 ± 0.7^ fg^	11.7 ± 0.9^cde^	12.8 ± 0.9^de^	157.00 ± 6.56^ g^	141.67 ± 8.02^hi^	90.20 ± 1.58^abc^	28.37 ± 0.50^ef^
K20	78.5 ± 0.9^ cd^	11.5 ± 1.5^cde^	9.5 ± 0.4^ h^	174.33 ± 6.66^bcde^	157.00 ± 5.57^cdef^	90.07 ± 1.26^abc^	29.37 ± 0.70^cde^
WT	70.4 ± 3.3^gh^	14.1 ± 1.2^a^	15.3 ± 2.6^ab^	167.20 ± 10.63^ef^	151.9 ± 9.85^efg^	90.85 ± 1.53^abc^	29.53 ± 0.75^cde^

Different lowercase letters in the same column of data indicate significant differences (P < 0.05), and the same or no letters indicate no significant difference (*P* > 0.05)

**Table 2:** Agronomic traits of wild-type rice

Name	Plant height(cm)	Panicle length(cm)	Tiller number	Total panicle grains	Panicle grains	Seed setting rate(%)	Thousand–grain weight(g)
WT1	68.2	12.5	23	178	165	92.7	30.16
WT2	80.2	16.3	12	150	137	91.33	28.48
WT3	73.1	13.8	18	162	148	91.36	29.28
WT4	71.4	12.7	19	168	154	91.67	29.74
WT5	72.5	13.5	20	169	150	88.76	29.4
WT6	68	15.8	21	159	145	91.19	28.9
WT7	67.5	12.3	23	160	145	90.63	28.93
WT8	72.2	15.9	19	192	170	88.54	31.2
WT9	69.3	12.8	21	155	143	92.26	28.55
WT10	71.5	15.3	20	169	153	90.53	29.4
WT11	68	15.1	21	175	161	92	30.2
WT12	68.3	14.3	22	169	151	89.35	29.7
WT13	69.5	14.7	21	179	161	89.94	30.5
WT14	69.8	14.9	20	154	141	91.56	28.3
WT15	73.6	13	18	160	142	88.75	29.5
WT16	74.5	14.1	19	183	170	92.9	30.3
WT17	67.9	12.9	23	164	153	93.29	29.1
WT18	68.1	14.5	21	161	145	90.06	29.9
WT19	66.1	13.8	24	175	161	92	30.1
WT20	68.9	13.6	21	162	143	88.27	28.94
error	3.3	1.2	2.6	10.63	9.85	1.53	0.75
average value	70.4	14.1	20.3	167.2	151.9	90.85	29.53

### Measurement of peroxidase activity

**Fig. 4:**
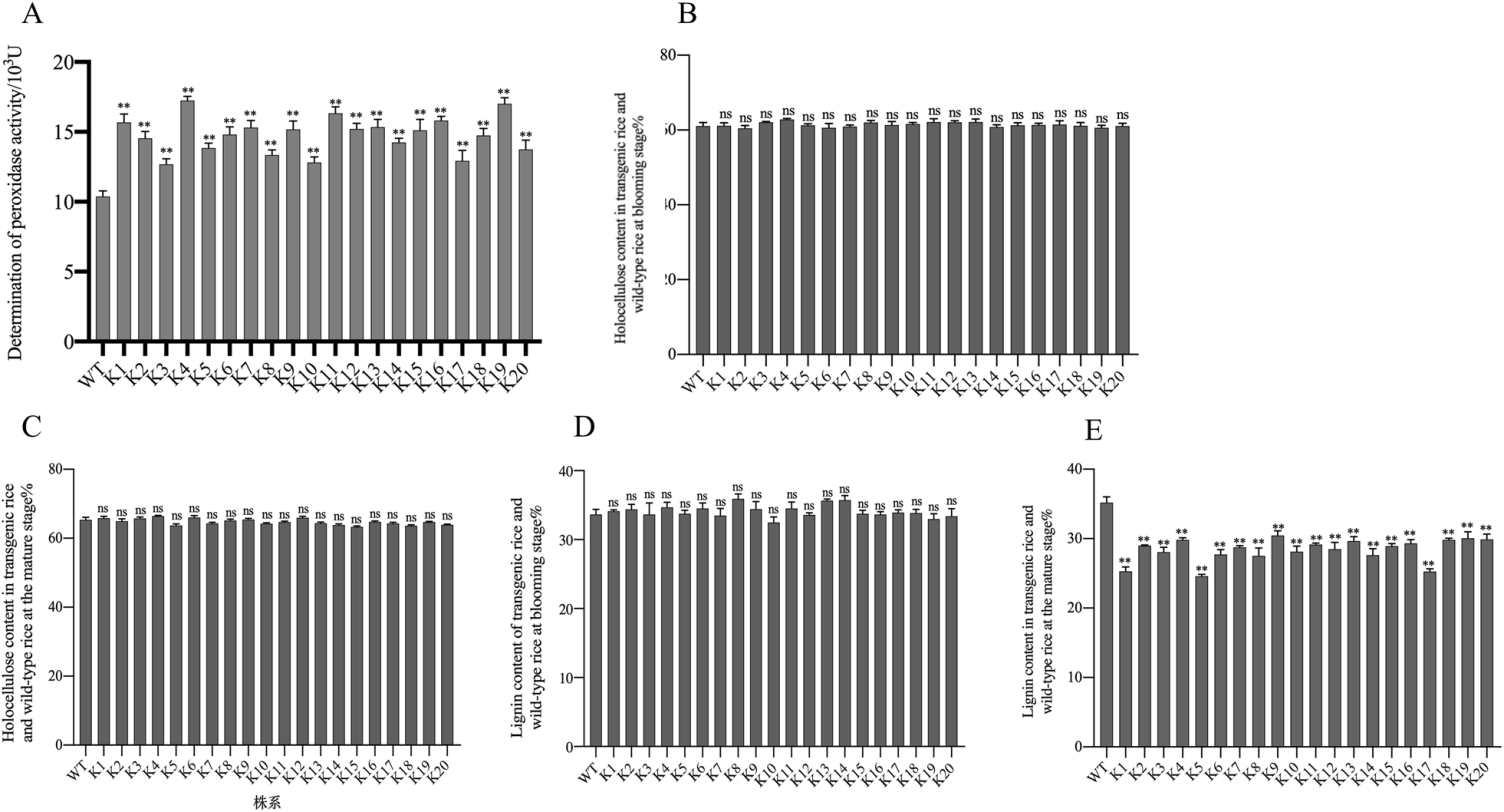
Physiological and biochemical measurements. **A** Detection of peroxidase activity in transgenic maize plants. **B** Holocellulose content in transgenic rice and wild-type rice at the blooming stage. **C** Holocellulose content in transgenic rice and wild-type rice at the mature stage. **D** Lignin content of transgenic rice and wild-type rice at the blooming stage. **E** Lignin content in transgenic rice and wild-type rice at the mature stage

Peroxidase activity was detected spectrophotometrically in transgenic plants, and these plants were identified through PCR-based screening using wild-type rice plants as a control. Transgenic rice leaves demonstrated higher peroxidase activity than the control leaves, but the activity levels varied notably among the transgenic rice leaves (Fig. 4A). According to SPSS analysis, the peroxidase activities of wild-type rice and transgenic rice lines were extremely different *(P* < *0.01*). Figure 4B shows that the peroxidase activity in the leaves of the transgenic rice at the mature stage was higher than that in the wild-type rice, and there were also certain differences between the different transgenic lines. Compared with the wild type, the highest value of peroxidase activity of the transgenic rice line K4 increased by 65.38%, and the lowest value of K3 increased by 22.16% compared with the wild type. The results showed that the exogenous genes *SHP* and *GLOX* can be expressed normally and efficiently under the guidance of the promoter, and the peroxidase activity of the transgenic rice plants was significantly increased.

### Determination of heterocellulose content

The heterocellulose content is the sum of the cellulose and hemicellulose contents. However, when cellulose and hemicellulose contents are measured separately, a partial loss in yield is witnessed, which results in an inaccurate measurement of lignin components. This inaccuracy can be eliminated by measuring the heterocellulose content. In this study, the holocellulose contents of rice in the flowering stage are shown in Fig. 4B. The holocellulose content of wild-type rice was 61.12% ± 0.89%, the highest holocellulose content in transgenic rice lines was observed in K4, which was 62.85% ± 0.28%, and the lowest holocellulose content was in K2, which was 60.48% ± 0.74%. Data analysis using SPSS 26.0 showed that the cellulose content in wild-type rice lines was not significantly different from that of transgenic rice lines (*P* > *0.05*), indicating that the introduction of the exogenous genes *SHP* and *GLOX* did not affect the synthesis of cellulose in plants during blooming. The total cellulose content of rice at the mature stage is shown in Fig. 4C. The holocellulose content of wild-type rice was 63.35% ± 0.78%, the highest holocellulose content of transgenic rice lines was found in K6, which was 66.04% ± 0.87%, and the lowest holocellulose content was in K15, at 63.30% ± 0.52%. Data analysis using SPSS 26.0 showed that the cellulose content in wild-type rice lines was not significantly different from that of transgenic rice lines (*P* > *0.05*), indicating that the expression of the exogenous genes *SHP* and *GLOX* did not affect the synthesis of cellulose in plants at the mature stage.

### Determination of lignin content

The lignin content of rice in the flowering stage is shown in Fig. 4D. According to measurements, the lignin content of wild-type rice lines was 33.63% ± 0.76%, the highest lignin content of transgenic rice lines was observed in K8, which was 35.90% ± 0.70%, and the lowest lignin content of transgenic rice lines was found in K10, at 32.47. % ± 0.81%. Using SPSS 26.0 for data analysis, the lignin content of wild-type rice lines was not significantly different from that of transgenic rice lines (*P* > *0.05*), and the lignin content of some transgenic rice lines was slightly higher than that of wild-type rice. Studies have shown that the exogenous gene *SHP* can promote the synthesis of lignin in the early stage of plant development and can effectively degrade lignin under the action of H_2_O_2_ in the later stage. Therefore, it is speculated that the expression of the exogenous gene *SHP* does not affect lignin synthesis in the early stage of the plant and may also promote lignin synthesis.

**Fig. 5:**
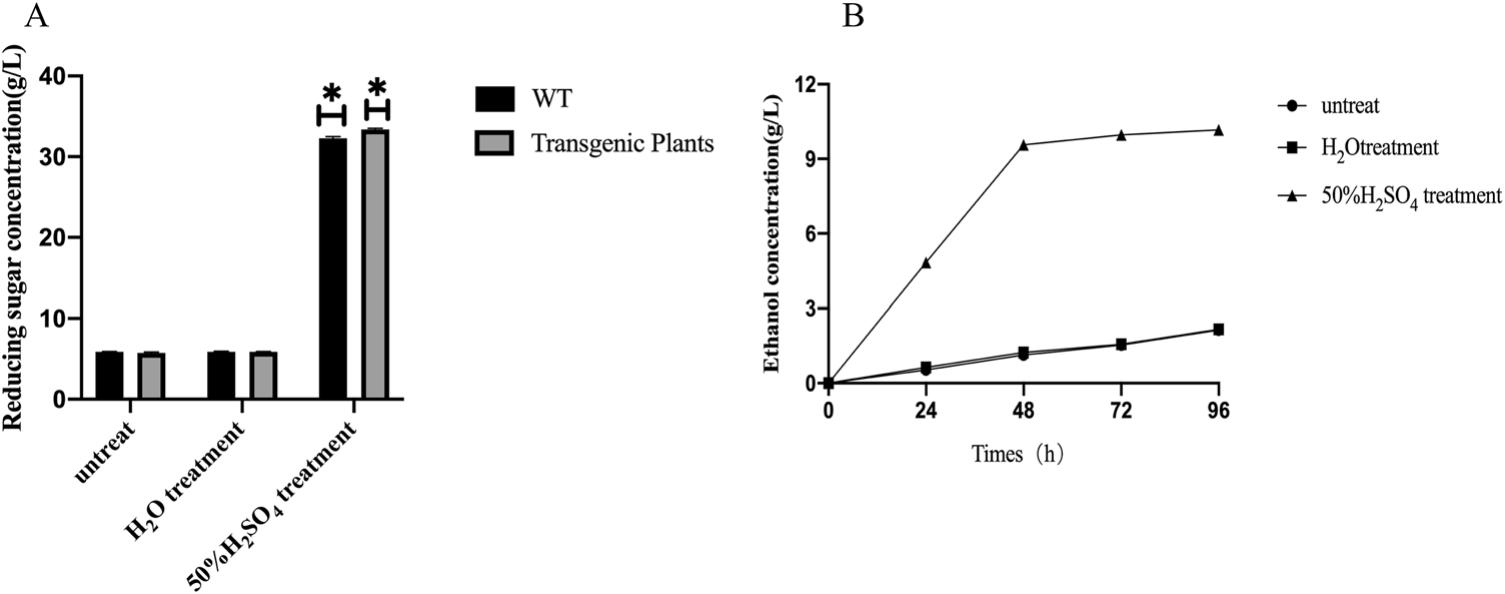
Ethanol fermentation experiments using transgenic plants. **A** Hydrolytic reducing sugar content. **B** Ethanol concentration during fermentation

The lignin content of rice in the mature stage is shown in Fig. 4E. According to the measurements, the lignin content of the wild-type rice lines was 35.17% ± 0.84%. The highest lignin content of the transgenic rice lines was found in K9, which was 30.45% ± 0.66%, and the lowest lignin content of the transgenic rice lines was found in K5, which was 24.58. % ± 0.27%. Using SPSS 26.0 for data analysis, the lignin content of wild-type rice lines was significantly different from that of transgenic rice lines (*P* < *0.01*). It can be seen from Fig. 5E that the lignin content of transgenic rice at the maturity stage was lower than that of wild-type rice, and there were also certain differences between different transgenic lines. Compared with the wild type, the highest lignin content of the transgenic rice line K9 was reduced by 13.42%, and the lowest value, that of K5, was reduced by 30.11% compared with that of the wild type. The results showed that *GLOX* can provide H_2_O_2_ for *SHP*, and *SHP* can effectively degrade lignin under the action of H_2_O_2_. Therefore, it is speculated that the expression and interaction of the exogenous genes *SHP* and *GLOX* can effectively reduce the lignin content.

### Results of ethanol fermentation experiments using transgenic plants

#### Determination of reducing sugar contents

The reducing sugar concentration determined after cellulase hydrolysis is shown in Fig. 5A. The experimental data results represent three groups of duplicates. In the no pretreatment group and the ddH_2_O treatment group, the contents of reducing sugars obtained from cellulase-degraded transgenic rice and the control (WT) were essentially the same. However, after 50% sulfuric acid pretreatment, both the transgenic rice and the control showed an increase in the content of hydrolysed sugars (23.2 g/L); this was significantly higher (*p* < *0.05*) than that in the untreated group (5.8 g/L).

#### Determination of ethanol contents

Ethanol fermentation experiments were performed on ddH_2_O-treated transgenic rice, 50% sulfuric acid-pretreated rice, and nonpretreated rice. Compared with the same fermentation conditions, the three prefermentation treatments resulted in changes in the amount of ethanol produced. Under an initial fermentation pH of 5.0 and temperature of 30 °C and an inoculation amount of 10%, fermentation products were obtained at 24, 48, 72, and 96 h to determine the ethanol concentration. As shown in Fig. 5B, the ethanol content determined at each period of pretreatment with 50% sulfuric acid was much higher than the corresponding value of the untreated group and ddH_2_O pretreatment. The final ethanol concentration of transgenic rice (9.1 g/L) was significantly higher than that of nontransgenic rice (2.6 g/L) but slightly lower than the concentration of the acid pretreatment group (9.9 g/L).

## Discussion

The expression vector plays a crucial role in transgene expression [19]. In this study, we constructed a K167 backbone vector from pCAMBIA3301, which contained herbicide and kanamycin resistance genes. The K167 vector contained a β-glucuronidase gene in the T-DNA region of the SHP-GLOX gene, harbouring the GRP peptide. The gene cassette in the K167 vector is under the control of the constitutive CaMV 35S promoter, which is active throughout plant development [20]. Thus, in this study, we used CaMV 35S to enhance *SHP* and *GLOX* gene expression in rice plants.

Rice is a vital food crop worldwide. The main objective of rice research is genetic engineering-mediated improvement of the overall characteristics and resistance to biotic and abiotic stress through genetic engineering [21]. *Agrobacterium*-mediated transgenic technology is widely used in the research and development of rice plants [22]. It is influenced by a myriad of factors, such as embryo pretreatment, concentration and time of infection, temperature and time of coculture, and concentration of screening agents [23–25].

In this study, rice was transfected with the *SHP* and *GLOX* genes using Agrobacterium-mediated transformation. Large young rice embryos with vigorous growth were selected for this transformation using phosphinothricin as the screening agent. In the screening stage, the calli appeared brown in colour, and at the end of the screening stage, resistant calli appeared yellowish in colour with a fluffy texture and strong regenerative capacity [7, 26]. A total of 380 seeds were transformed in the experiment. After a series of transformations, a total of 21 transformation events were obtained, and the transformation efficiency was 5.53%.

Generally, a high DNA and RNA concentration with low RNA and protein contaminants is a prerequisite for southern hybridization. Herein, the phenol–chloroform extraction method was used to remove impurities, such as polysaccharides and proteins; in addition, RNase A was used to reduce RNA contaminants from DNA precipitate after dissolution [27]. In conventional southern blotting, radioisotopes are used to label probes that have potential safety hazards and require proper handling under specialized laboratory conditions. The currently used highly symplectic labelling method is safe and efficient for probe labelling. Southern hybridization can be used to detect foreign genes in a genome and evaluate species homology [28]. The commonly used RNA extraction reagents include TRIzol, sodium dodecyl sulfate (SDS), and CTAB. As shown in a previous comparative analysis of several RNA extraction methods, the SDS extraction method yielded the highest RNA purity and integrity [29].

In this study, we did not observe any significant phenotypic differences between transgenic and wild-type rice plants. There was a significant difference (*P* < *0.01*) in the control peroxidase activity in transgenic rice, with the maximum increase compared to the control up to 65.38%. It is speculated that the exogenous genes SHP and GLOX can be expressed normally and efficiently under the guidance of the promoter, and the peroxidase activity of the transgenic rice plants can be significantly improved. There was no significant difference in the cellulose content in the transgenic rice lines at the flowering and maturity stages compared with that of the wild type (*P* > *0.05*). It is speculated that the introduction of the exogenous genes SHP and GLOX has no significant effect on the synthesis of plant cellulose and hemicellulose. The lignin content of transgenic rice lines during the blooming stage was not significantly different from that of the wild type (*P* > *0.05*). The lignin content of rice at the mature stage was determined, and it was found that the contents of the transgenic rice lines were significantly different from that of the wild type (*P* < *0.01*), and the lignin content of the transgenic rice lines was lower than that of the control group. It is inferred that the exogenous gene GLOX is expressed at full maturity. At the beginning of the period, it was expressed and provided H_2_O_2_ for SHP, which may have participated in the process of lignin depolymerization and reduced the lignin content of the plant. The commonly used methods to determine lignin content are acetic acid, acid washing, and Klason methods [30]. To determine the lignin content, these three methods were compared by determining the saccharification efficiency of various *Miscanthus* cultivars. The Klason method was found to be the most effective and suitable method for high-throughput analyses [18]. This result suggested that *SHP* expression promoted lignin synthesis as well as lignin degradation in rice plants with GLOX expression.

Lignin has a complex structure, and it degrades slowly under natural conditions. Conventional physical and chemical methods used for lignin degradation have a low efficiency of lignin removal, culminating in environmental pollution [31]. Current strategies for lignin removal have been primarily focused on the synthesis of lignin monomers, and the genes associated with this process are being studied in most plants; however, other approaches, such as lignin polymerization, have not been extensively investigated [32]. Peroxidase and laccase promote the polymerization of lignin monomers [28, 33] as well as lignin degradation under certain conditions [28, 36]. Restricted genetic diversity can supply only a limited number of elite genes for modern plant cultivation and transgenesis [37]. Thus, peroxidases are expected to enhance lignin degradation in transgenic crops for the effective utilization of biomass resources [38].

## Conclusion

This research has created a rice breeding material with normal growth and yield but stalks that are more likely to be degraded in the later stage to breed rice varieties whose stalks are easily used for energy. Our results will improve the industrial and commercial applications of rice straw.

## Methods

### K167 plasmid optimization

*Escherichia coli* DH5α cells were procured from TransGen Biotech (Beijing, China). The pCXUN plasmid and plant expression vector pCAMBIA3301-glycine-rich protein (GRP)-SHP-GLOX (K167) used in this study were taken from our laboratory stocks. The GRP protein guides the localization of the SHP-GLOX fusion protein to the plant cell wall in peptide-producing rice [14]. K167 contains the constitutively expressed active cauliflower mosaic virus (CaMV) 35S promoter, which is widely used to generate transgenic plant varieties.

Total RNA was extracted from soybean hulls, and then reverse transcription of RNA was performed by reverse transcription polymerase chain reaction technology to obtain first-strand cDNA. Using the synthesized cDNA as a template, the SHP gene was amplified by PCR. To express the SHP gene in rice, a recombinant vector was constructed on the basis of the pCAMBIA3301 vector. First, SHP was connected to the pMD-20 T vector, and then the vector strains pNMCS-ZSAG12p-GRP-GLOX-NOS, pMD 20-T-GRP0.9, pMD 20-SHP and pCAMBIA3301 were cloned and stored in the laboratory. After screening and identification, a new recombinant plasmid named the GRP-SHP-GLOX expression vector (K167) was obtained for the production of transgenic rice (Fig. 1C). (The specific construction process is in Annex 1).

### Genetic transformation of rice

Competent *Agrobacterium* cells were prepared from *Agrobacterium tumefaciens* EHA105 colonies cultured on YEB medium. The cells were transformed using the electroporation method, and the transformants were identified using PCR. The transformed *A. tumefaciens* cells were preserved using a 20% glycerol solution and stored at − 80 °C. The *Oryza sativa* L. spp. *japonica* rice lines were transformed with the K167 vector using *Agrobacterium* strain EHA105 [15].

### Identification of transgenic rice by PCR

Genomic DNA was extracted from leaves of transgenic rice plants using the cetyltrimethylammonium bromide (CTAB) method. The extracted DNA was subjected to PCR-based screening with the SHP and Bar primers.

### Southern blot analysis

#### Preparation of digoxigenin (DIG)-dUTP-labelled probe

The *Bar* gene in the rice genome was amplified using PCR. The amplified PCR product was visualized using AGE and recovered through the gel extraction procedure and was later denatured in boiling water for 10 min. The denatured PCR product was transferred to an ice bath for 2–3 min, and 4 μl of DIG-High Prime was added to it. This mixture was incubated overnight at 37 °C, and the reaction was terminated by heating at 65 °C for 10 min. The labelled probe was stored at 4 °C until further use.

#### Southern blotting

The rice genome was digested overnight at 37 °C with a restriction enzyme, *HindIII* (New England Biolabs), in a reaction containing 30 μl of genomic DNA extracted from the rice leaves, 5 μl of *HindIII*, 10 μl of buffer, and 55 μl of ddH_2_O. The digested product was resolved on a 1% agarose gel for 24 h at 25 V. The agarose gel was later transferred to a nylon membrane, washed with 2X saline–sodium citrate, blotted dry, and placed in a transparent Ziploc bag. The DNA was fixed on the membrane by ultraviolet irradiation for 5 min, and the membrane was stored at 4 °C until use.

In a bag, the nylon membrane was treated with 10 ml of preheated (37 °C water bath) hybridization solution. Prehybridization was performed at 37 °C with shaking at 80 rpm for 30 min. Five microlitres of *Bar* probe was denatured for 5 min, cooled in an ice bath, and centrifuged at 12,000 rpm for 30 s. Then, 500 μl of hybridization solution was added, and the mixture was incubated overnight at 37 °C with shaking at 80 rpm. The film was washed and developed as per the standard protocol [16]. Briefly, the membrane was transferred to a new bag, washed gently with light shaking at 65 °C, blocked for 30 min at room temperature (24 °C), and incubated with the antibody solution (1:10,000 dilution) for 30 min at room temperature. After washing, the membrane was equilibrated in the assay solution for 2–5 min followed by incubation with the test solution for 5 min. This membrane was developed by exposing it to X-ray film, the membrane hybridized with a digoxigenin-labelled probe overnight was observed and detected using the digoxigenin luminescence detection procedure with the Roche DIG High Prime DNA Labelling and Detection Starter Kit II (Roche Applied Science, Penzberg, Upper Bavaria, Germany) according to the manufacturer’s instructions.

#### RT–qPCR

RNA was extracted from fresh rice leaves using a plant RNA extraction kit (Kangwei Century Biotech Co., Taizhou City, China) according to the manufacturer’s instructions. The diluted cDNA was used as a PCR template. G*lyceraldehyde 3-phosphate dehydrogenase* (*GAPDH*) was used as a reference gene. The sequences of the primers are shown in Table 3.

**Table 3:** Name and sequence of primer

Name	Sequence of primer5′ → 3′	TM ( C)
SHP–F	AGCTTCTGTTCTGGGAGGAGGTC	61.1
SHP–R	AGGTTGAAGAAAGGTGCTGGAAGG	59.6
GLOX–F	CGACCCGCCGTTCATGTTCAG	62
GLOX–R	GACTTGGACCTTGCTCGCCTTC	61.3
18 s rRNA–F	CTACGTCCCTGCCCTTTGTACA	59.5
18 s rRNA–R	ACACTTCACCGGACCATTCAA	55.6

#### Basta smear

A 1:5000 dilution of 10% Basta solution (Bio Basic, Markham, ON, Canada) was prepared using deionized water. Transgenic rice leaves were treated with this diluted Basta solution, and changes at the site of application were evaluated after 8–10 days.

#### Biochemical analysis of transgenic rice plants

##### Agronomic traits

Twenty well-growing mature and control rice plants were randomly selected, and their plant height, panicle length, effective tiller number, total grain number, real grain number, seed setting rate per plant and 1000-seed weight were measured.

##### Peroxidase activity assay

Crude enzyme extract was prepared from leaves of transgenic rice, and the peroxidase activity in this extract was evaluated spectrophotometrically (Shanghai Jingke Industry Co., Ltd). A unit of enzyme activity was calculated as an increase of 0.01 light absorption units per minute (i.e., change in absorbance at 470 nm/raw weight).

##### Determination of hemicellulose content

The hemicellulose content of transgenic rice in the flowering stage and the maturity stage was determined using the NaClO_2_ method, as described previously (GB2677.10–1995) [17].

Determination of lignin content

The lignin content in transgenic plants in the flowering stage and the maturity stage was determined using the Klason method [18].

##### Ethanol fermentation of different genotype plants

Stems of the same parts of the transgenic and control plants at the mature stage were cut into sections of approximately 3 cm and divided into three groups. The first group was immersed in ddH_2_O in a 45 °C water bath for 1 h. The second group was treated with 50% sulfuric acid and hydrolysed at 120 °C for 1.5 h. The third group was not treated. After hydrolysis, the solution was neutralized to pH 5.0 with sodium hydroxide. The stem sections of the two groups were placed in an oven for drying (100 °C), ground into a powder, and then sieved through a 40-mesh sieve. Then, acetic acid-sodium acetate cellulase solution (pH 4.8) was added, and the solution was immersed in a water bath at 37 °C for 24 h. The DNS (dinitrosalicylic acid) method was used to determine reducing sugars. Reducing sugars were used as a carbon source after the rice was hydrolysed; the fermentation medium was added at a solid–liquid ratio of 1:10 and sterilized at 121 °C for 20 min. Preserved *Saccharomyces cerevisiae* was isolated from the slant culture medium and cultured in a seed medium at 28 °C with shaking at 200 rpm for 16 h and inoculated at a 10% inoculation amount. Under fermentation at pH 5.0 and 30 °C, the products of fermentation at 24, 48, 72, and 96 h were obtained for the detection of the ethanol content.

## Data Availability

All data generated from this study are included in this published article and supporting materials.
